# Sustainable and Cost-Effective Gel Documentation

**DOI:** 10.3390/mps6020021

**Published:** 2023-02-21

**Authors:** Nadeem Asad, Scott Cregg, Sudeep Shakya, Sutton Stegman, Lisa Timmons

**Affiliations:** 1 Department of Molecular Biosciences, The University of Kansas, 1200 Sunnyside Avenue, Lawrence, KS 66045, USA; 2 Department of Biochemistry and Molecular Medicine, University of West Virginia Health Science Center, 64 Medical Center Drive, Morgantown, WV 26506, USA

**Keywords:** DNA electrophoresis, protein electrophoresis, gel documentation, lab sustainability

## Abstract

A common laboratory method involves gel electrophoresis followed by photographic documentation of the results, a procedure which is performed worldwide by students and experienced scientists alike. Proprietary Gel Documentation Systems are convenient and useful for documentation of electrophoresis results, but the systems can be prohibitively expensive to purchase and repair, they contain features that are not necessary for everyday documentation, and some users may not find the systems intuitive to operate. We describe our gel documentation setup that meets the everyday needs for documentation in our lab. The setup is inexpensive, modular, user friendly, and increases sustainability through extending the working life of obsolete cell phones, iPads, or other electronic devices containing a camera. More importantly, the setup completely shields users from potentially damaging ultraviolet radiation.

## 1. Introduction

Gel electrophoresis is a separation and analysis technique that is commonly used in most research institutions, especially those with a molecular biology, biochemistry, genetics, or forensic focus. Polyacrylamide gel electrophoresis is used in analysis of proteins, low molecular weight polynucleotides, or small amounts of radiolabeled polynucleotides, while agarose gel electrophoresis is commonly used in analysis of DNA and RNA [1,2,3,4,5]. After separation, the biopolymers in the gel can be stained using specific colorimetric or fluorescent dyes for documentation purposes or downstream processes. Individual protein bands can be revealed using Coomassie stains, while RNA or DNA can be observed using base intercalating molecules such as ethidium bromide, which displays concentration- and orientation-dependent fluorescence upon binding [6,7,8,9,10].

Photographing gels has evolved from the use of silver emulsion film to instant film (Polaroid) photography to image capture by digital cameras. Gel Documentation Systems have been developed and evolved to meet electronic capture and documentation needs of laboratories, as gel electrophoresis is performed multiple times per day in many labs. However, these systems are expensive, contain integrated components that make repair difficult and costly, and can occupy a large footprint on the lab bench. Many clever adaptations of existing parts and equipment toward less expensive gel documentation systems have been described [11,12,13,14,15,16]. With the expanded reach of consumer devices with cameras, do-it-yourself approaches have become increasingly enticing, yet such efforts can result in inconsistent imaging or exposure to hazardous ultraviolet (UV) radiation.

Here, we describe a gel documentation setup we developed after the breakdown of a previous system. The system is modular in nature: all parts are built or repurposed and are easily replaced or upgraded, helping provide insurance against high replacement costs in the event of future breakdowns. The system was developed with sustainability, safety, and simplicity in mind, and it meets the everyday needs for gel documentation in our lab.

## 2. Materials and Equipment

(The suggestions below regarding transilluminating light boxes may be impacted by enactments of proposed rules from the Department of Energy in the U.S., which will phase out mercury-containing fluorescent and compact fluorescent bulbs in favor of LED lighting [17]. Mercury reduction is a world-wide trend, with 137 countries in the 2021 Minamata Convention on Mercury agreeing to phase out categories of fluorescent bulbs by 2025. This may result in a market surge of inexpensive used equipment. Fortunately, there are LED replacement lamps for all types of fluorescent bulbs, and retrofit kits are available for all products without the need for re-wiring [18]. Many LED products are direct retrofits. However, as mentioned below, there are currently no direct-replacement LED substitutes in the high energy UV ranges.)

**Figure 1:**
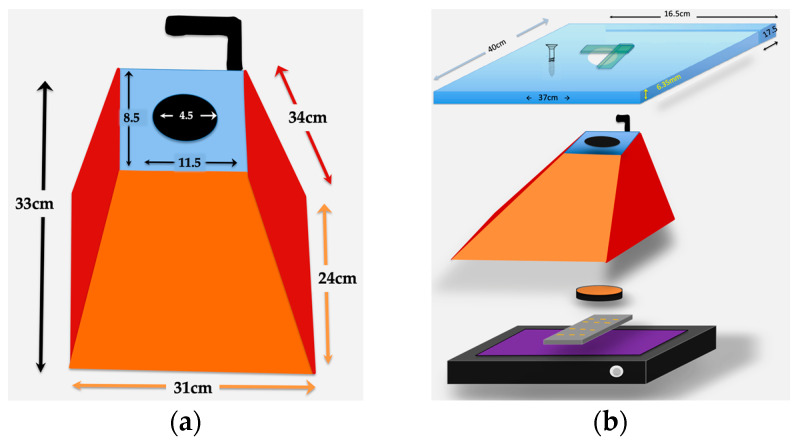
Dimensions and assembly of hood and camera mount. (**a**) Dimensions of the repurposed Fotodyne photography hood are provided for those who choose to build one. This version, 33 cm in height, completely covers the UV light source, has a metal camera mount fastened to the back (black L-shape), and has a 4.5 cm diameter hole for a lens mount. (**b**) Order and assembly of parts for the phone/iPad stand. A grooved line ~4 mm wide was cut into the quarter-inch acrylic sheet at the indicated position, ending ~1.5 cm from the lens holder. This was done to accommodate the camera mount, see Figure 2. The acrylic sheet was fastened into the top of the photography hood using 4 screws. A barrier filter (590 nm for ethidium bromide staining) is placed underneath the hole, at the top of the photography hood, covering the hole. This was taped in place for easier removal when photography of protein gels is performed or when shifting bandpass filters for SYBR-stained gels A layer of plastic wrap can be used to protect the surface of the UV transilluminator during imaging of wet gels, although care must be taken that the brand of plastic does not absorb UV.

**Figure 2:**
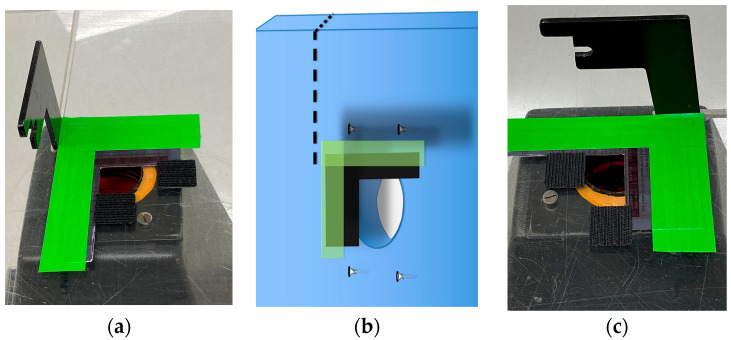
Details of adapting the camera mount for cell phones or iPad. Because of the large size of the camera hole, we placed an L-shaped piece of heavy black paper over the top right corner of the hole and secured the paper with green labeling tape. (**a**) The arm of the camera mount is positioned to the left of the green tape which is securing the black paper to the hood. (**b**) A diagram of the photo in (**a**) shows the acrylic sheet in blue, and the dotted line is the position of the cutout for the camera mount. The black paper and green tape cover three of the four fastening screws; (**c**) is a photo from a different angle.

**Figure 3:**
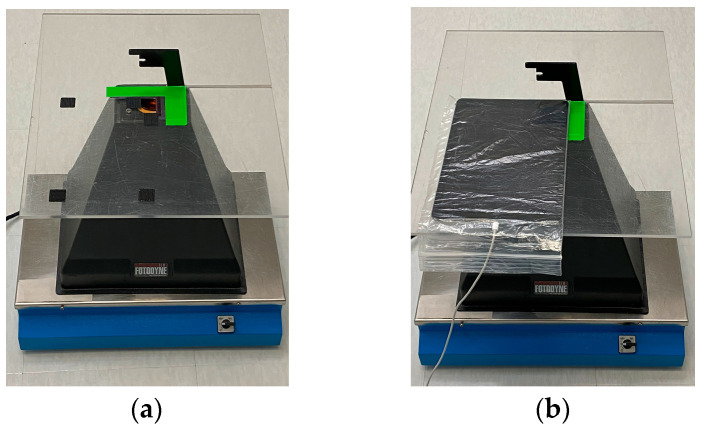
Fully assembled and operational camera mount and hood. A transparent, sealable plastic bag was used as a protective cover for the iPad. First, a ~2 cm square of plastic was removed from one side in the top corner of the bag so that the camera lens would not be covered by the plastic. This cutout faces down on the acrylic sheet, with the iPad resting on top. The iPad is pushed into the top right corner of the bag, with the camera facing down. The best position is determined empirically using an ethidium-stained gel as guide, and the position of the camera is marked on the acrylic. (**a**) When the optimal position is determined, hook-and-loop fasteners with adhesive backing are placed on the back of the plastic bag, the adhesive covering is removed from the other side, and the bag, with iPad, is positioned correctly on the acrylic camera mount, leaving the hook and loop fasteners attached in position. These fasteners are also visible in Figure 2. (**b**) The gel documentation system with discontinued iPad mounted is shown. The bag is sealed around the cord, providing protection from aqueous solutions. Excess bag material can be taped together or sealed with a cooking pouch sealer to minimize movement of the iPad within the bag. This arrangement has proven quite secure. The photography hood can be moved using the camera mount as a handle, and the iPad stays securely mounted on the hood.

- Free standing UV transilluminating box (for documenting gels with ethidium bromide).
  1. Costs for a transilluminator vary from $900–$2500 USD. The cost is lower if purchased from a supplier of used scientific equipment, with some suppliers offering warranties. Gel boxes are long-lasting pieces of equipment and a good investment. Our lab’s UV light box, a Cole-Parmer 97500 series, was inherited from a dismantled lab, and is 30+ years old, requiring only a change of bulbs and fluorescent bulb starters. For safety and performance reasons, the UV light box should be obtained from a reputable manufacturer or scientific supplier (see Discussion). Repurposing a light box with UV bulbs is not recommended due to cost and safety concerns.
  2. Consider all DNA detection dyes that will be used by the lab and ensure that the excitation wavelengths for the dyes are able to pass through the transillumination surface plate. The more expensive, all-in-one Gel Documentation Systems provide flexibility regarding dye usage, as multiple excitation wavelengths are possible and multiple bandpass filter sets are provided with the instrument. Our lab uses dilute ethidium bromide to stain gels in routine genotyping or quality and quantity checking, and SYBR gold when downstream applications require extraction of stained DNA [9,10]. Ethidium bromide’s fluorescence excitation peak, 312 nm [19], is a standard excitation wavelength for UV transilluminating boxes. SYBR gold has a blue light excitation peak at 495 nm, and a second, weaker excitation band at ~300 nm, energies emitted from a UV transilluminator [20]. While LED diodes with 312 nm emission are available, there are currently no off-the-shelf replacements for mercury fluorescent bulbs in boxes optimized for 312 nm excitation, and many bulbs marketed to the public as UV or germicidal do not actually emit UV below 400 nm.
  3. If both UV and blue light illumination (for SYBR) are desired, and if UV transilluminator wavelengths are not sufficient for SYBR (due to blockage of blue wavelengths), blue light converter screens have been developed (Syngene). These are placed on top of the UV transilluminator and convert UV to blue light. Alternatively, an additional box can be configured for SYBR dyes. A separate dual blue/white light box can also be useful for detection of Coomassie stained protein gels.
  4. For UV transilluminators that pass wavelengths under 495 nm, imaging SYBR gels without incurring UV-induced damage to the DNA is possible by placing an acrylic sheet underneath the gel. Acrylic (PMMA poly(methyl methacrylate) or Plexiglas^®^ Perfexion™ UF-5 blocks 95% of UV (Plexiglas U825-UVA: [21]). Small sheets of UV-resistant acrylic are sold as glass substitutes for picture frames; larger sizes can be purchased from a plastics manufacturer (EMCO industrial plastics).
  5. UV protection goggles. The photography hood covers the entire illumination plate, protecting users from UV during photography, but when excising DNA from gels, the plastic cover and UV goggles should be used. Opaque paper towels should cover exposed areas of the UV light box and users should also wear UV protection goggles.
- White light illumination box for photography of protein gels
  1. These are relatively inexpensive, when new, and can be obtained from photography or art suppliers. Newer equipment is outfitted with LED lamps.
  2. Blue LED bulbs (495 mn) are suitable for SYBR gold excitation. Newer gel boxes are outfitted with both white and blue lamps for viewing Coomassie stained protein gels or SYBR-stained DNA gels.
  3. Care should be taken to avoid placing wet gels on the surface of the light box. They are not water-tight, and Coomassie stain can build up on the surface.
- Light shielding photography hood. The simple Fotodyne photography hood we describe here is difficult to find as new equipment. The hoods can be purchased from suppliers of used equipment for $10–$80 USD. With the dimensions provided in Figure 1A, a similar hood could easily be constructed using lightweight wood or plywood. The purpose of the hood is to shield the experiment from light, protect the user from UV, and provide a stable platform for the camera. Other shapes of hoods will likely work as well. An appropriately-sized Styrofoam shipping box might serve well—it would be waterproof, and a sturdy strip of plastic, such as a ruler, could be glued to its back wall to serve as a handle. A coating of matte black paint or spray paint on the Styrofoam would improve image quality.
- Bandpass filters: 590 nm for ethidium bromide; 530/40 nm for SYBR gold.
- Obsolete electronics with camera. The device should be wiped clean of data and apps and removed from online visibility. Power and data transfer cords are needed. Images here used a 1st gen iPad Air with 5 MP camera and 9.7 in display.
- Transparent, sealable plastic bag to protect the electronics.
- Camera stand. As described, we built a platform from an acrylic sheet using woodworking tools. The sheet provides a stable, hands-free support for the camera during photography. The dimensions of the camera stand we built are in Figure 1, with additional details in Figure 2.
- Data storage. A secure method should be devised to easily transfer images to working laboratory computers or cloud storage.
- Squares of hook-and-loop style fasteners (Velcro^®^ brand) with adhesive backing.
- The component parts are put together as described in Figure 1, Figure 2 and Figure 3.

## 3. Results

With the photography hood covering the entire transillumination plate of the UV box, users are safely shielded from ultraviolet radiation during gel imaging. Fastening the acrylic sheet into the top surface of the hood with a screw allows for stability, as does the cutout made in the acrylic, to accommodate the attached camera mount. The camera mount is a convenient handle, allowing the hood to be easily lifted as gels are placed on or removed from the UV box. The iPad is firmly attached to the acrylic sheet using hook and loop adhesive strips; therefore, moving or jostling the hood does not affect the position of the camera. The choice of a transparent acrylic sheet as a camera support over an opaque material, such as wood, allows us to easily work with materials on the lab bench, below the documentation system, as gels are being photographed. An opaque material would cause the documentation system to appear larger and cumbersome. We fashioned our camera support from a scrap sheet of acrylic, which proved to be a convenient size. Using a smaller acrylic square would likely be equally effective but, given the size and weight of the iPad, we did not reduce the size of the acrylic as the system seems well-balanced when the iPad is mounted.

**Figure 4:**
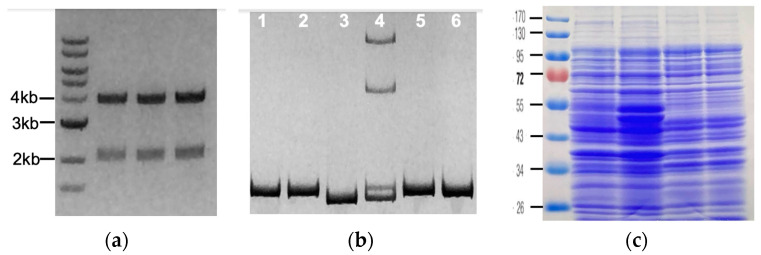
Examples of images obtained using our gel documentation apparatus and the autofocus feature of the iPad. The brightness of the images was adjusted for cosmetic reasons and the images were cropped; otherwise; no other image manipulations were performed. (**a**) Restriction digests of 3 plasmids were loaded on a 1% agarose/TAE gel next to molecular weight marker, with the MW of 3 bands indicated. (**b**) Low molecular weight PCR products from genotyping experiments were resolved on a 15% polyacrylamide/TBE gel. The DNA in lanes 1 and 2 is 9 base pairs longer than the DNA in lane 3; Samples 1, 2, 5 and 6 contained wild type DNA; sample 3 was obtained from a homozygous mutant; sample 4 is heterozygous. (**c**) Homogenized yeast samples were separated on a 12% polyacrylamide/SDS gel and Coomassie- stained for protein. Samples in (**a**,**b**) were run in the presence of ethidium bromide and photographed using a UV light box; the gel in (**c**) was photographed on a regular light box.

**Figure 5:**
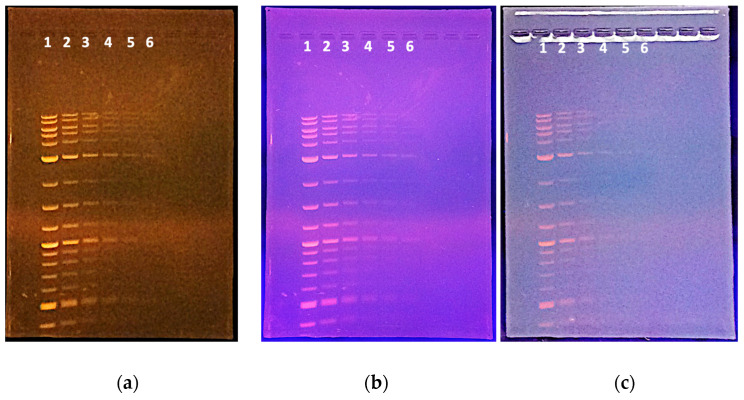
Sensitivity of documentation system using a 5 MP iPad Air camera. A 1:2 dilution series of a DNA molecular weight standard (NE Biolabs 1 kb plus ladder) was loaded on a 1% agarose/TAE gel, with 10 uL loaded per lane and six samples loaded. The amount of DNA in each lane is indicated in Table 1. The same gel was imaged under three conditions: (**a**) standard photo with UV illumination; (**b**) a photo without the 590 nm bandpass filter, and (**c**) a photo taken without using the UV-shielding hood—this is not advised due to the potential for eye damage; (**c**) demonstrates that the quality of image capture is much reduced without a light-shielding hood as fewer low abundance bands are visible.

The camera in the obsolete iPad is sufficient for the everyday documentation needs of the lab, as demonstrated in Figure 4. The images nicely reproduce the results, and we can obtain quantitative information from the images as well (Figure 5 and Table 1). The iPad Air is easy to use—the plastic bag covering the device does not prevent the touch pad from working, and no keyboard typing is needed to image or transfer files. Undergraduates new to the lab need few instructions to master imaging. Even without image enhancements or adjustments, the 5 MP camera easily photographs 1.3 ng of longer DNA molecules. Smaller DNA fragments, <4 ng or less, are also captured (Figure 5 and Table 1). This is well within the detection limit of ethidium bromide.

**Figure 6:**
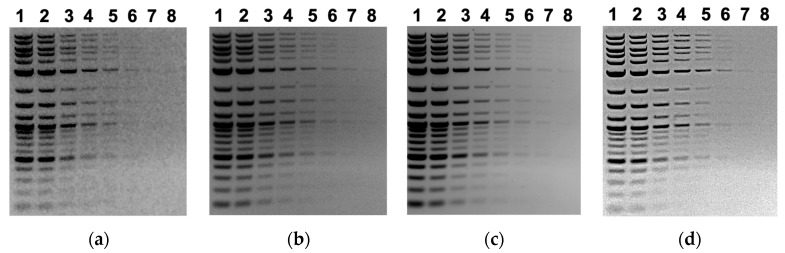
Comparison of images obtained from our repurposed documentation system and other gel documentation system packages. The images are photographs of the same gel obtained using (**a**) our system, using a repurposed 5 MP iPad Air; (**b**) using an AlphaImager EP (Alpha Innotech, ~$2000 USD used); (**c**) Azure Biosystem C400 ($41,000 USD new from VWR); and (**d**) using our system and a newer, non-obsolete iPhone 12. A 1:2 dilution series of a DNA molecular weight standard (NE Biolabs 1 kb plus ladder) was loaded on a 1% agarose/TAE gel, with 10 uL loaded per lane and eight samples loaded, as in Table 1. Because of the wide variation in light level capture, each image was processed using Photoshop to produce an image with best visibility of lower concentration bands.

We compared our repurposed camera system to existing Gel Documentation Systems by obtaining image files of the same gel. As seen in Figure 6, the more expensive Gel Documentation Systems, even older models (Figure 6b), produced higher quality images than our system (Figure 1a). However, in situations where visualization of low yield bands is important, we can easily improve the quality of our image capture by replacing the repurposed iPad with a newer device such as a cell phone (Figure 1b, compare with Figure 1a). As the device’s camera is contained within transparent plastic, this temporary replacement can be performed quickly and safely without contamination of the owner’s phone with laboratory solutions. For our needs, the slight increase in ability to visualize low concentration bands does not justify the cost (or larger footprint) of a new documentation system, especially since other spectrophotometric devices can more accurately determine concentrations of low yield samples.

## 4. Discussion

The idea of using electricity to separate biomolecules has taken many forms as the field of electrophoresis has evolved from its initial, Nobel Prize-worthy experiments. In the 1930s, Arne Tiselius used electricity to separate proteins in solution using U-shaped vessels [22,23]. Separation of molecules in a semi-solid media followed soon afterward and involved the use of potato starch in “zone” electrophoresis [24,25]. The development of a polyacrylamide matrix for separating proteins in cylindrical tubes [1,25], and the development of refined agar products as an inert separation matrix [26] were more immediate predecessors of the gel electrophoresis performed today. Gel electrophoresis is now ubiquitous and is one of the “top ten” vitally important separation and analysis techniques taught to beginning researchers [27]. The global electrophoresis market, valued at 2.3 billion USD in 2021, is predicted to grow to 3.8 billion by 2030, with increases driven by advances in capillary electrophoresis, by increased use of electrophoresis in forensics and detection of cancer, transmitted disease, and genetic disorders, and by growth of science and science teaching in developing countries [28]. Like DNA sequencing, PCR, and spectrophotometry, gel electrophoresis is a staple and routine technique. The added value for improvements in cost or efficiency would be multiplicative for this expansively used technique.

Good lab practice and experimental reproducibility require proper documentation of experimental results. In the 1930s, photography was essential for both documentation and detection in electrophoresis experiments, as proteins undergoing separation were detected visually and photographically based on schlieren light refraction patterns produced by the electrophoretic separation and concentration of proteins in transparent solutions [29,30,31,32]. Today, many colorimetric stains and fluorescent dyes are available that can be used to easily detect proteins in gels [7]. Photography continues to improve as well. Since the sale of the first digital camera and Adobe Photoshop 1.0 image-manipulation program in 1990, digital photography has advanced at an unrelenting pace.

Digital cameras are now integrated into many devices, which soon become unusable due to technical advances and a corresponding lack of support and replacement parts, or planned obsolescence. Disposal of electronic devices that have reached the end of their operable lives have produced a global e-waste problem, threatening human health and the environment. Extending the life of electronic devices, through repurposing, is one solution to slowing the pace of e-waste growth.

Here, we document that the camera in a 5 MP first-generation iPad Air, no longer supported by Apple, provides sufficient resolution to meet the gel documentation needs of a working lab, and our adaptations extend the working life of the device. Our photography setup allows us to easily test other devices; for example, we also obtained excellent gel images using an iPhone 11 and other older cell phones, including Android. With this setup, students at all levels, including high school students in the US, require no training to image the results of their electrophoresis experiments or to transfer their images to permanent electronic storage and notebooks. Our system is multi-user friendly. All members of the lab and guests can quickly obtain electronic files of images or upload the image files to cloud storage. While we have not encountered a need to move our device, it can be easily dismantled and reassembled to a different bench or room—the portability of our device is limited by the size and weight of the illumination box. Fashioning a smaller hood with a smaller transillumination box would allow for greater portability, if required.

An important consideration when using cameras from obsolete electronic devices is the lack of original equipment manufacturer updates that can affect the security of the devices. Personal data in obsolete devices should be securely erased, with non-camera apps removed, and use of the device should be restricted to taking photos. Informational technology units in businesses and universities will not allow unsupported devices to be connected to their campus networks. Using short-range wireless technology such as Bluetooth is one convenient way to transfer selected images to a working lab computer. A USB or other appropriate cable in most situations is a safer means of securely importing specific images from the disconnected device directly to a secure lab folder.

By using obsolete smart phones or iPads, the most expensive aspect of our gel documentation system is the transillumination box. A UV light box is required for ethidium bromide staining, which is still commonly used in electrophoresis due it its low cost and long shelf life. Considered moderately toxic, using ethidium bromide in a restricted work area and with proper precautions and clean up, its hazards to health and environment can be easily minimized [33].

A UV light box is best purchased from a scientific supplier due to safety issues and functionality. The most important feature of the UV light box is the ability of its surface plate to transmit UV radiation, and minimal visible light, from the UV source. The spectral properties of the surface plate not only enhance the fluorescence from DNA bands but also prevent an image of the glowing UV bulb from appearing in the gel photo. The surface plate is the most expensive part of a UV light box and is difficult for an individual to source inexpensively at a specific size or shape. It might be possible to repurpose a UV light box surface plate from a non-operational UV light box if a similar sized visible light box is available and the light box can handle the wattage of UV bulbs. However, extreme care must be taken to ensure there are absolutely no light leaks due to the potential for eye damage from UV exposure. As repurposing a UV light box would likely not be a simple undertaking for most biology labs, it is safer and more expedient to purchase a UV light box. UV light boxes can remain operational for 30+ years or longer, provided the wiring remains intact. The fix for some “broken” UV light boxes simply involves replacing UV bulbs or the fluorescent bulb starter.

Cell phones and iPads produce image files that are easily processed using open source and commercial software such as GIMP, Photoshop, ImageJ, or Preview. We have not encountered a need for file conversion, and images are easily cropped, re-sized, inverted, labeled, contrast enhanced, and renamed so that the original unmodified files remain readily available. Using a Mac computer, we set up a workflow using Automator to perform routine manipulations such as cropping, color removal, and size reductions on images to help manage the file size of electronic lab notebooks.

The details we provide here of successfully repurposed equipment using obsolete electronic devices highlight our labs’ increasing efforts toward laboratory sustainability and environmental responsibility. Our adaptations were inexpensive, and our system is easy and safe to use, as the user is entirely shielded from UV light when ethidium bromide is used for detection. Because our documentation setup is modular, parts, such as the camera, can be easily and inexpensively substituted.

## Figures and Tables

**Table 1:** Amount of DNA in each band (Figure 5). A dilution series was made using a DNA molecular weight marker (NE Biolabs 1 kb plus ladder) and the amount of DNA present in each band is listed below. Bolded rows indicate DNA at higher relative concentrations.

	Lane 1	Lane 2	Lane 3	Lane 4	Lane 5	Lane 6
Length	Mass (ng)	Mass (ng)	Mass (ng)	Mass (ng)	Mass (ng)	Mass (ng)
10 kb	40	20.0	10.0	5.0	2.5	1.3
8 kb	40	20.0	10.0	5.0	2.5	1.3
6 kb	48	24.0	12.0	6.0	3.0	1.5
5 kb	40	20.0	10.0	5.0	2.5	1.3
4 kb	32	16.0	8.0	4.0	2.0	1.0
**3 kb**	**120**	**60.0**	**30.0**	**15.0**	**7.5**	**3.8**
2 kb	40	20.0	10.0	5.0	2.5	1.3
1.5 kb	57	28.5	14.3	7.1	3.6	1.8
1.2 kb	45	22.5	11.3	5.6	2.8	1.4
**1 kb**	**122**	**61.0**	**30.5**	**15.3**	**7.6**	**3.8**
0.9 kb	34	17.0	8.5	4.3	2.1	1.1
0.8 kb	31	15.5	7.8	3.9	1.9	1.0
0.7 kb	27	13.5	6.8	3.4	1.7	0.8
0.6 kb	23	11.5	5.8	2.9	1.4	0.7
**0.5 kb**	**124**	**62.0**	**31.0**	**15.5**	**7.8**	**3.9**
0.4 kb	49	24.5	12.3	6.1	3.1	1.5
0.3 kb	37	18.5	9.3	4.6	2.3	1.2
0.2 kb	32	16.0	8.0	4.0	2.0	1.0
0.1 kb	61	30.5	15.3	7.6	3.8	1.9

## Data Availability

Not applicable.
